# The Association Between Adherence to the Dutch Healthy Diet Index and Glaucoma Prevalence—The Maastricht Study

**DOI:** 10.3390/nu18091360

**Published:** 2026-04-25

**Authors:** Yu Yu, Tos T. J. M. Berendschot, Simone J. P. M. Eussen, Carla J. H. van der Kallen, Carroll A. B. Webers, Wishal D. Ramdas

**Affiliations:** 1 University Eye Clinic Maastricht, Maastricht University Medical Centre+, 6229 HX Maastricht, The Netherlands; t.berendschot@maastrichtuniversity.nl (T.T.J.M.B.);; 2 Mental Health and Neuroscience Research Institute, Maastricht University, 6200 MD Maastricht, The Netherlands; 3 Institute of Nutrition and Translational Research in Metabolism, Maastricht University, 6229 ER Maastricht, The Netherlands; 4 Department of Epidemiology, Maastricht University, 6200 MD Maastricht, The Netherlands; simone.eussen@maastrichtuniversity.nl; 5 Cardiovascular Research Institute Maastricht (CARIM), Maastricht University, 6229 ER Maastricht, The Netherlands; 6 Department of Internal Medicine, Maastricht University Medical Centre+, 6229 HX Maastricht, The Netherlands; 7 Department of Ophthalmology, Erasmus MC University Medical Center, 3000 CA Rotterdam, The Netherlands

**Keywords:** glaucoma, dietary patterns, Dutch Healthy Diet Index, intraocular pressure, epidemiology

## Abstract

**Objectives:** To investigate the association between adherence to national nutritional guidelines (Dutch Healthy Diet Index [DHD-index]) and glaucoma prevalence and to explore whether this association changed after accounting for measured intraocular pressure (IOP). **Methods:** This cross-sectional study used baseline data 2010–2013 from The Maastricht Study, a population-based cohort in The Netherlands. Adults aged 40–75 years with implausible dietary intake were excluded. Dietary intake was evaluated using a validated food frequency questionnaire, and adherence was quantified by the DHD-index. All participants underwent ophthalmic examination including perimetry and IOP measurement. Logistic and linear regression models examined associations of DHD adherence with glaucoma prevalence and IOP. Additional exploratory analyses assessed whether the association with glaucoma was attenuated after accounting for measured IOP. **Results:** Among 5729 participants (mean age: 59.5 ± 8.7 years; 50.1% female), glaucoma prevalence was 9.7% (n = 558). Each 10-point increase in DHD-index score was associated with 12.5% lower odds of glaucoma prevalence (odds ratio [OR]: 0.88; 95% confidence interval [CI], 0.83 to 0.93) and lower IOP (β: −0.17; 95% CI, −0.25 to −0.09 mmHg). Individuals in the highest DHD adherence tertile had 38% lower odds of glaucoma than those in the lowest tertile (OR 0.62; 95% CI, 0.50 to 0.76). Additional adjustment for measured IOP yielded similar estimates. **Conclusions:** Higher adherence to the Dutch Healthy Diet was associated with a lower glaucoma prevalence. The association was only minimally attenuated after accounting for measured IOP. Longitudinal studies should examine whether adherence to national dietary guidelines is associated with glaucoma onset and progression.

## 1. Introduction

Glaucoma is the leading cause of irreversible blindness, characterized by progressive optic neuropathy and retinal ganglion cell degeneration [1,2]. While intraocular pressure (IOP) is the only modifiable risk factor, a substantial proportion of glaucoma patients continue to experience vision loss despite adequate IOP control [3]. This suggests that neurodegenerative mechanisms independent of IOP contribute significantly to disease progression [3,4].

The role of diet in neurodegenerative diseases has been extensively studied, particularly in Alzheimer’s disease and Parkinson’s disease [5], where dietary patterns such as the Mediterranean diet [5,6] and DASH (Dietary Approaches to Stop Hypertension) Diet [5] have shown significant neuroprotective effects. Given the shared embryonic origin of the eye and brain [7], the neuroprotective effects observed in nutritional epidemiology on the central nervous system are likely to also extend to the optic nerve [7], highlighting the potential role for dietary interventions in glaucoma. However, most existing glaucoma research has focused on isolated nutrients—such as flavonoids (e.g., found in tea, berries, and citrus fruits) [8], nitrate-rich foods (e.g., leafy greens) [9], and omega-3 fatty acids (e.g., from fish and nuts) [10]—that influence ocular blood flow, inflammation, and oxidative stress. While these findings offer mechanistic insights, they do not capture the interactive and cumulative effects of overall dietary patterns [11].

Previous studies on dietary patterns, like the Mediterranean, DASH and MIND (Mediterranean–DASH Intervention for Neurodegenerative Delay) diets [12], have demonstrated promising neuroprotective effects. However, these dietary patterns do not always align with habitual food consumption in all populations. The Dutch Healthy Diet (DHD) Index 2015 [13] is a validated measure of adherence to the 2015 Dutch Dietary Guidelines. These food-based guidelines were developed from systematic reviews and meta-analyses relating diet to major chronic diseases and broadly promote a more plant-based and less animal-based dietary pattern [14]. Greater adherence to these guidelines has previously been associated with lower all-cause mortality and lower risk of several non-communicable diseases in the Rotterdam Study, supporting the DHD-index as a relevant measure of overall diet quality in a Dutch population [15].

This study investigates the association between DHD adherence and glaucoma prevalence, with particular attention to IOP-dependent and IOP-independent pathways that may help explain this association. By focusing on nation-specific, evidence-based dietary guidelines, this study aims to strengthen the evidence base on dietary quality in glaucoma and to inform future studies on whether dietary patterns may be relevant to glaucoma prevention.

## 2. Materials and Methods

### 2.1. Study Design and Population

**Figure 1:**
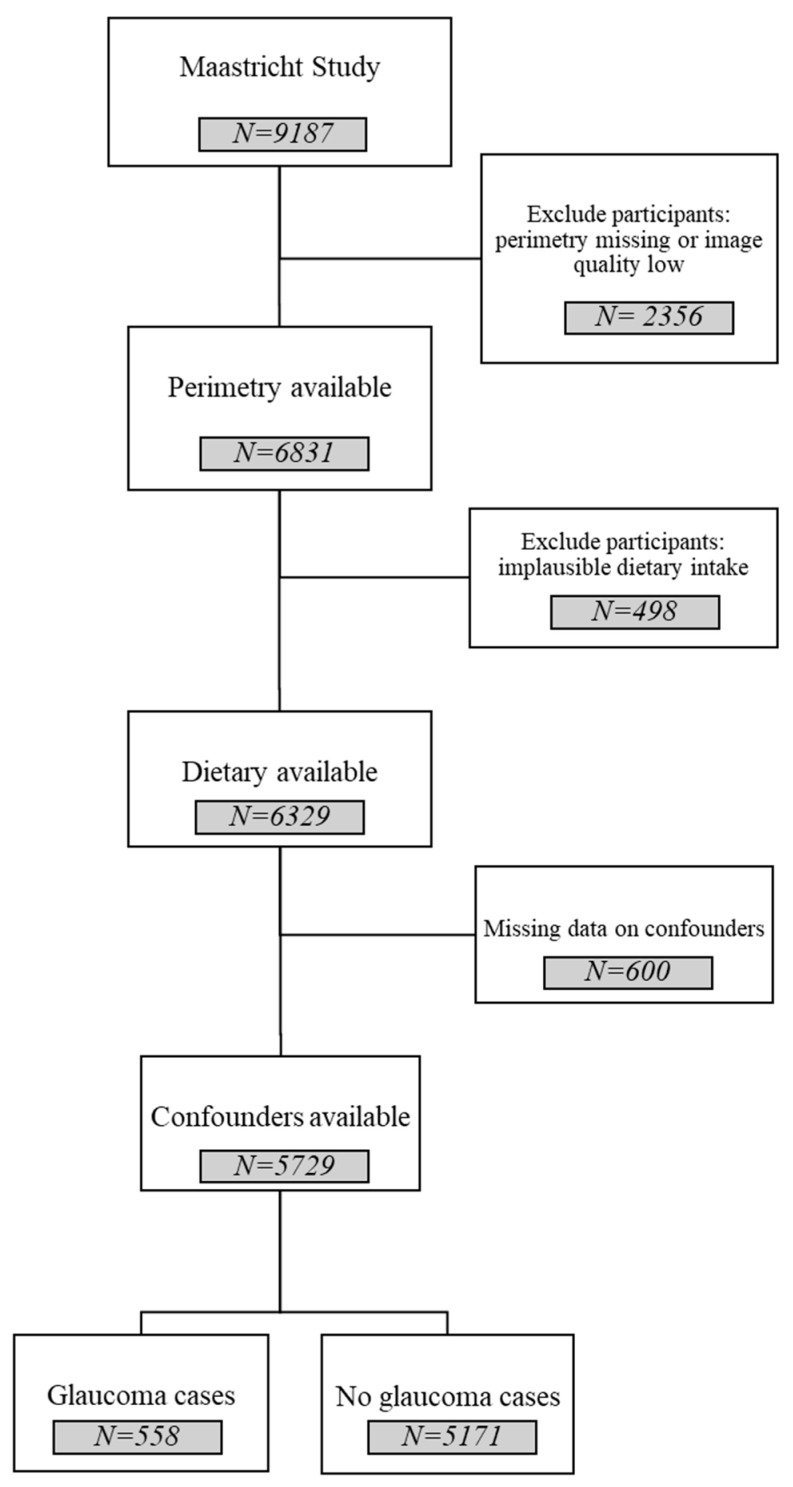
Flow diagram for selection and inclusion of population.

This study used baseline data from The Maastricht Study, an ongoing population-based cohort in which individuals with type 2 diabetes mellitus were oversampled to improve efficiency and statistical power for comparisons by diabetes status. The rationale and methodology of the study have been described in detail elsewhere [16]. Briefly, The Maastricht Study is designed to investigate the etiology, pathophysiology, complications, and comorbidities of type 2 diabetes through extensive phenotyping. Participants were aged 40 to 75 years and resided in the southern Netherlands (province of Limburg). The present analysis includes cross-sectional data from 6831 participants who completed the baseline examination between November 2010 and October 2013. After prespecified exclusions (Figure 1), 5729 participants remained for the analytic sample. Recruitment was conducted using mass media campaigns and direct mailings, based on municipal registries and the regional Diabetes Patient Registry, with stratification by diabetes status. The study protocol was approved by the institutional medical ethics committee (NL31329.068.10) and the Dutch Ministry of Health, Welfare, and Sport (permit 131088-105234-PG). All participants provided written informed consent following the Declaration of Helsinki to participate in the study.

### 2.2. Glaucoma Case Ascertainment

Glaucoma classification was performed by three independent glaucoma specialists based on visual field testing with the Heidelberg Edge Perimeter (HEP; Heidelberg Engineering, Heidelberg, Germany). Mean retinal sensitivity was also assessed with the HEP, as described previously [17]. Visual fields labeled as ‘Outside Normal Limits’ by the Glaucoma Hemifield Test were independently reviewed and categorized as confirmed glaucoma, suspected glaucoma, or non-glaucoma by the three specialists (CW, RC, MC). During this adjudication process, fundus photographs and OCT scans were also reviewed to exclude alternative non-glaucomatous causes of visual field loss, including hemianopic defects, high myopia-related field loss, and other fundus abnormalities. To ensure consistency and minimize interobserver variability, an initial consensus meeting was held. Cases with discordant assessment were subjected to secondary review and additional consensus discussions. Remaining discrepancies were resolved through a final adjudication involving all available clinical data.

We did not apply the International Society of Geographical and Epidemiological Ophthalmology (ISGEO) case definition because it relies on population-specific percentile cutoffs for vertical cup-to-disc ratio (VCDR) and VCDR-asymmetry derived from individuals with normal visual fields, and such cutoffs cannot be assumed to be transferable across study populations. Accordingly, fixed quantitative structural thresholds, such as VCDR cutoffs, were not prespecified. Rather, structural information from fundus photographs and OCT scans was incorporated as part of the specialist adjudication process. Further procedural details are provided in the Supplementary Materials.

### 2.3. Assessment of Intraocular Pressure (IOP)

Intraocular pressure was measured using an automated tonometer (TONOREF II, NIDEK Co., Ltd., Gamagori, Japan). Three consecutive IOP measurements were obtained from each eye, and the mean value of these measurements was recorded for each eye separately. The final IOP recorded for each participant represented the average of the mean IOP values from both eyes. Importantly, and consistent with contemporary evidence that glaucoma can arise at normal IOP and may progress despite IOP control, IOP values were not incorporated into the glaucoma definition [4,18]. For participants receiving IOP-lowering medication, measured IOP values were adjusted by dividing by a factor of 0.7. Additionally, for participants who reported a prior history of glaucoma surgery, the recorded IOP values were standardized to 30 mmHg [4,19,20,21,22].

### 2.4. Dietary Assessment

Diet was assessed at baseline by a validated, self-administered food frequency questionnaire (FFQ) [23]. More details on the development and the validity of this FFQ have been reported elsewhere [24]. The questionnaire captured the frequency and portion size of each food item consumed. Participants with implausible dietary intake were excluded, defined as total energy intake <800 or >4200 kcal/day for men, and <500 or >3500 kcal/day for women.

Adherence to the 2015 Dutch dietary guidelines was quantified using the Dutch Healthy Diet (DHD) Index 2015 [13], a 15-component diet-quality score developed to operationalize the Dutch food-based dietary guidelines. Each component is scored from 0 to 10 according to the degree of adherence to the guideline threshold, yielding a total score from 0 to 150 points (0–140 points in this study because data on filtered coffee were unavailable). Higher scores indicate better overall diet quality. Detailed component definitions and scoring thresholds are provided in the Supplementary Materials.

### 2.5. Assessment of Covariates

Covariates that were extracted from the electronic database and general questionnaire [16] included age; sex; education level (low, middle, or high); smoking status (never, current, or former smoker); alcohol consumption (g/day); total energy intake (kcal/day); physical activity (h/week); glucose metabolism status (normal glucose metabolism, prediabetes, or type 2 diabetes) based on fasting venous plasma glucose samples (mmol/L), 2 h post-load glucose samples (mmol/L), and glucose-lowering medication use; as well as BMI (kg/m^2^); office blood pressure (mmHg) during a physical examination; hypertension (yes/no). Participants were classified as having hypertension if the average systolic blood pressure was ≥140 mmHg, or the average diastolic blood pressure was ≥90 mmHg, or they used antihypertensive medication. Further details on covariate assessment and definitions are provided in the Supplementary Materials.

### 2.6. Statistical Analysis

Between-group differences were assessed using independent t-tests for continuous variables and chi-square tests for categorical variables. For skewed continuous variables, Mann–Whitney U tests were applied as appropriate.

The DHD scores were analyzed both as a continuous variable (per 10-point increase) and as a categorical variable based on tertiles: Tertile 1 (low adherence), Tertile 2 (middle adherence), and Tertile 3 (high adherence). Participant characteristics across DHD tertiles were compared using one-way ANOVA for continuous variables and chi-squared tests for categorical variables.

Multivariable logistic regression was used to examine the association between DHD adherence and glaucoma prevalence. Odds ratio (OR) with corresponding 95% confidence interval (CI) were estimated for both continuous and tertile-based DHD exposures. To assess linear trend across tertiles, the median DHD score within each tertile was assigned to all participants in that tertile and entered into the regression model as a continuous variable. Linear regression models were applied to assess the association between DHD-index and IOP, with results reported as β-coefficients with corresponding 95% CI per 10-point increase in DHD score.

Three models were prespecified. Model 1 was adjusted for age, sex, and total energy intake. Model 2 was further adjusted for BMI, physical activity, smoking status, and educational attainment. Model 3 included all covariates in Model 1 plus hypertension and glucose metabolism status. These models were used to examine the association under different adjustment sets and were interpreted as complementary rather than strictly sequential models. Accordingly, Models 2 and 3 are presented as complementary models reflecting lifestyle-related and cardiometabolic factors, respectively, rather than as a single progression toward one fully adjusted model. As a supplementary analysis, each lifestyle-related covariate included in Model 2 (BMI, physical activity, smoking status, and educational attainment) was entered individually into Model 1 to assess the change in the DHD effect estimate.

To examine whether the association between DHD-index and glaucoma prevalence changed after accounting for measured IOP, we fitted an additional logistic regression model by adding IOP to Model 1. This analysis was intended to examine attenuation after accounting for IOP, rather than to serve as a formal mediation model.

We also conducted an exploratory cross-sectional mediation analysis to assess whether measured IOP statistically accounted for part of the observed association. Both the mediator and outcome models were adjusted for age, sex, and total energy intake, corresponding to Model 1, to retain a parsimonious baseline specification. Given the cross-sectional design, these analyses were used to explore possible patterns in the data and were not intended to establish temporal sequence or causality. For interpretability, we report an OR-scale decomposition of the indirect, direct, and total effects using a product-of-coefficients approach, with 95% CI derived from 5000 nonparametric bootstrap resamples. See Supplementary Materials for model specification and estimation details.

Sampling weights were not applied. As sensitivity analyses, we compared glaucoma prevalence after exclusion of participants with cardiovascular disorders (e.g., type 2 diabetes, hypertension, or lipid disorders). As an additional sensitivity analysis of possible reverse causation, we repeated the main glaucoma prevalence analyses after excluding participants with indicators of glaucoma treatment or related eye disease history. We also tested for effect modification by diabetes status by including an interaction term between DHD adherence and diabetes status in Models 1–3.

All statistical analyses were performed using SPSS v28.0 (SPSS Inc., Chicago, IL, USA) and R v4.4.1 (R Foundation for Statistical Computing, Vienna, Austria) with packages dplyr, broom, mediation, haven, ggplot2. A *p*-value < 0.05 was considered statistically significant.

## 3. Results

### 3.1. Selection and Characteristics of the Study Population

Following the application of all exclusion criteria (Figure 1), Table 1 summarizes the characteristics of glaucoma cases (n = 558) and non-cases (n = 5171). Notably, participants diagnosed with glaucoma were significantly older, had higher BMI, and reported lower alcohol consumption than non-cases. Furthermore, the glaucoma group had higher proportions of type 2 diabetes, hypertension, and smoking. As anticipated, the IOP was significantly higher among individuals with glaucoma than among non-cases. Adherence to the DHD was significantly lower in glaucoma cases than non-cases.

### 3.2. Characteristics of Participants by Adherence to the DHD-Index (Per Tertiles)

Table 2 presents the characteristics of participants across tertiles of adherence to the DHD-index. Participants in the lowest adherence tertile had the highest prevalence of glaucoma, greater BMI, and an increased prevalence of type 2 diabetes and hypertension compared to those in the highest tertile. Moreover, individuals in Tertile 1 were more likely to be male, current smokers, and high alcohol consumers.

In contrast, participants in Tertile 3 had the highest proportion of females, a greater prevalence of normal glucose metabolism, and higher levels of educational attainment. This group also reported greater engagement in physical activity and had the lowest IOP.

### 3.3. Association Between DHD-Index and Glaucoma Prevalence

Table 3 shows that a higher DHD-index was significantly associated with a lower prevalence of glaucoma. In the multivariable-adjusted model (Model 1), each 10 score increase in DHD score (on a 0–140 scale) was associated with 12.5% lower odds of glaucoma prevalence (OR, 0.88; 95% CI: 0.83 to 0.93). In Model 2, the corresponding OR was 0.95 (95% CI: 0.89 to 1.02), and in Model 3, it was 0.89 (95% CI: 0.84 to 0.94). Thus, the association remained directionally consistent across models, although it was attenuated in Model 2. Component-specific associations are shown in Supplementary Figure S1.

Across the DHD tertiles, individuals in the highest adherence tertile (Tertile 3, DHD score > 91.2 points) had lower odds of glaucoma than those in the lowest tertile (Tertile 1, DHD score ≤ 77.6 points). In Model 1, participants in the highest tertile had 38% lower odds of glaucoma (OR, 0.62; 95% CI: 0.50 to 0.76). The corresponding ORs for Tertile 3 versus Tertile 1 were 0.85 (95% CI: 0.67 to 1.08) in Model 2 and 0.66 (95% CI: 0.53 to 0.81) in Model 3. Overall, the tertile-based analyses showed the same inverse pattern as the continuous analyses, with attenuation in Model 2.

In supplementary analyses adding each lifestyle-related covariate individually to Model 1, the association between a higher DHD score and lower glaucoma prevalence remained statistically significant in all four models (OR range, 0.88–0.91), with the greatest attenuation observed after adjustment for physical activity, followed by educational attainment and BMI, whereas adjustment for smoking had little impact (Table S1).

### 3.4. Association Between DHD-Index and IOP

We further investigated the association between adherence to the DHD and IOP. As shown in Table 3, multivariable-adjusted linear regression analyses indicated that each 10-point increase in the DHD score was significantly associated with a 0.17 (95% CI: −0.25 to −0.09) mmHg lower IOP in Model 1, which was similar to Model 2 and Model 3.

Examining the association across DHD tertiles, participants in Tertile 3 had significantly lower IOP compared to those in Tertile 1. In Model 1, the difference was −0.59 (95% CI: −0.88 to −0.31) mmHg, which was similar to Model 2 and Model 3.

### 3.5. Association Between DHD-Index and Glaucoma Prevalence After Adjustment for IOP

After adjustment for age, sex, and total energy intake, each 10-point increase in DHD score was associated with lower glaucoma prevalence 0.88 (95% CI: 0.83 to 0.93). Additional adjustment for IOP yielded similar estimates (OR, 0.88; 95% CI: 0.83 to 0.94), suggesting little attenuation after accounting for IOP.

**Figure 2:**
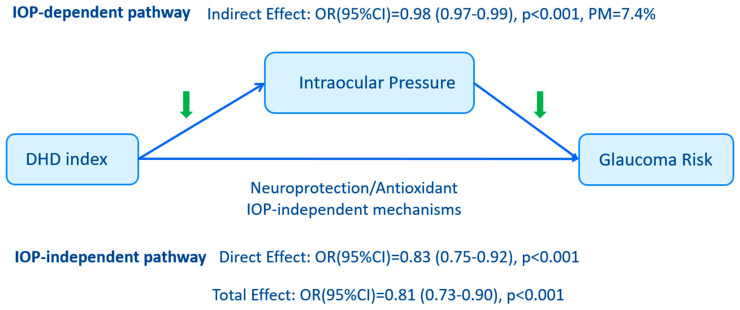
Exploratory cross-sectional decomposition of the association between DHD adherence, IOP and glaucoma prevalence. Notes: The results showed that for every 10-point increase in the DHD score, the odds ratio of glaucoma prevalence and its corresponding 95% confidence interval were presented. Models were adjusted for age, sex, total energy intake. Abbreviations: CI, confidence interval; PM, proportion mediated. Green downward arrows indicate the direction of the observed association along the IOP-related pathway.

Figure 2 summarizes the exploratory cross-sectional mediation analysis of IOP in the association between DHD-index and glaucoma prevalence. After adjustment for age, sex, and total energy intake, a 10-point increase in DHD score was associated with lower glaucoma prevalence (total effect OR, 0.81; 95% CI: 0.73 to 0.90). The indirect effect via IOP was small but statistically significant (OR, 0.98; 95% CI: 0.97 to 0.99), with the proportion mediated estimated at 7.4%. The corresponding direct effect estimate was 0.83 (95% CI: 0.75 to 0.92).

### 3.6. Sensitivity Analyses

In a sensitivity analysis excluding all participants with type 2 diabetes, glaucoma prevalence among participants with available glaucoma data changed only modestly, from 9.7% in the full analytic sample to 9.5% in the restricted sample. In separate sensitivity analyses, glaucoma prevalence was 9.1% after exclusion of participants with lipid disorders and 9.0% after exclusion of participants with hypertension.

In an additional sensitivity analysis excluding participants with indicators of glaucoma treatment or related eye history, the overall pattern of results was comparable to the primary analyses: the ORs for glaucoma per 10-point increase in DHD score were 0.87 (95% CI 0.82–0.92), 0.95 (95% CI 0.89–1.02), and 0.88 (95% CI 0.83–0.94) in Models 1–3, respectively.

In addition, no evidence of interaction between DHD adherence and diabetes status was observed in Models 1–3 (*p* for interaction = 0.93, 0.94, and 0.90, respectively); this suggests that the association did not differ materially by diabetes status.

## 4. Discussion

Higher adherence to the national Dutch dietary guidelines was associated with a lower prevalence of glaucoma in this cross-sectional sample. The association changed little after additional adjustment for IOP, suggesting that it was not fully explained by IOP alone. However, any interpretation in terms of IOP-dependent and IOP-independent mechanisms remains exploratory given the cross-sectional design.

This study highlights the value of evaluating adherence to evidence-based dietary guidelines in glaucoma research. Prior studies have examined the Mediterranean and MIND diets in relation to neurodegenerative diseases [5,6] including glaucoma [12,25], but most glaucoma-specific work has focused on single nutrients such as flavonoids, omega-3 fatty acids, and nitrates [8,9,10,26]. In contrast, assessing overall dietary quality—such as via DHD-index—captures the synergistic interactions among nutrients and provides a more holistic view of nutrition’s role in ocular health. This approach aligns with evidence suggesting that overall dietary patterns may better reflect cumulative and synergistic effects on oxidative stress, inflammation, and vascular dysfunction, all of which contribute to glaucoma pathogenesis [4,11,18,27]. On the DHD scale, a 10-point increase—approximately 7% of the attainable range in this study (0–140)—denotes a modest, achievable improvement in overall diet quality rather than a wholesale change; in practice, slightly more vegetables and fish and less processed meat and sugar-sweetened beverages/fruit juices would produce a change of this magnitude, consistent with Supplementary Figure S1 [13].

A further contribution of this study lies in its emphasis on national dietary guidelines. While diet scores such as the Mediterranean, MIND, and vegetarian diets have shown broad health benefits [25,28,29], their applicability between people from different cultures may be limited by differences in food preferences, access, and long-term adherence [28]. In contrast, the DHD-index is grounded in national dietary guidelines and was operationalized using intake data from commonly consumed foods in the Dutch population, including vegetables, dairy, tea, and vegetable oils [13]. Although the DHD uses a composite scoring approach similar to other indices (e.g., Mediterranean, DASH, and MIND) [30], its foundation in national dietary guidelines and incorporation of culturally specific recommendations may enhance its feasibility and relevance in the Dutch context [13,31]. We do not interpret the DHD-index as a fixed Dutch food pattern that should be exported unchanged to other populations. Rather, we view it as an example of guideline-based diet-quality framework that could be adapted to other settings using locally relevant foods and national dietary recommendation [13,31], although this was not examined in the present study. At the same time, our cohort was predominantly White (98%), and present effect estimates should therefore be generalized cautiously. Replication in more ethnically diverse cohorts, ideally using culturally adapted diet-quality indices, is needed before broader generalization can be made.

Recently, the Rotterdam Study reported no significant association between DHD adherence and incident open-angle glaucoma, whereas an inverse association was observed for the MIND diet [12]. Direct comparison with our findings is limited because dietary quality was operationalized and analyzed differently, including our use of a continuous DHD score and our additional analyses accounting for measured IOP. Differences in outcome definitions (incident vs. prevalent) and covariate adjustment strategies may also contribute to this discrepancy. Overall, the Rotterdam Study’s findings suggest that glaucoma risk may be more sensitive to specific dietary patterns than to broad guideline adherence alone.

One of our key findings was that higher DHD adherence was associated with lower IOP, consistent with studies linking dietary components—such as antioxidants, dietary fiber, and omega-3 fatty acids—to improved aqueous humor dynamics and trabecular meshwork function [10,31,32,33]. Specifically, antioxidants may reduce oxidative damage within the trabecular meshwork [32], thereby enhancing outflow facility [10]. These nutrients, abundant in DHD-recommended foods including vegetables, fruits, whole grains, legumes, fish, and tea, may contribute to ocular regulation both directly—via local tissue effects—and indirectly through systemic improvements in vascular function, insulin sensitivity, and reduced chronic inflammation [9,10,26,34]. These pathways are increasingly recognized as integral to IOP homeostasis [9,10,26,34].

Beyond its association with lower IOP, our findings are also compatible with the possibility that diet quality may be related to glaucoma through mechanisms not fully captured by measured IOP, although such interpretations remain exploratory in this cross-sectional study [35]. This is particularly relevant given that glaucoma can progress even when IOP is controlled [4,11]. Nutritional components abundant in the DHD—such as omega-3 fatty acids, B vitamins, flavonoids, and polyphenols [26]—have been linked to lower oxidative stress [27], reduced neuroinflammation [27], improved mitochondrial function [27], and reduced apoptosis [10] in experimental and observational studies. Although these findings are compatible with a partial IOP-related pathway, the association with IOP was small relative to normal physiologic variation and should not be interpreted as a clinically meaningful IOP-lowering effect at the individual level. Likewise, the small proportion mediated suggests that IOP accounts for only a limited part of the observed association.

To better understand the attenuation in Model 2, we examined each lifestyle-related covariate individually. The inverse association between higher DHD adherence and lower glaucoma prevalence remained statistically significant in all four analyses, and no single covariate accounted for the attenuation seen in the full lifestyle model. The greatest attenuation was observed after adjustment for physical activity, with smaller changes after adjustment for education attainment and BMI, whereas smoking had little influence. Taken together, these findings suggest that the weaker estimate in Model 2 reflects the combined conditioning on several correlated lifestyle factors rather than one dominant covariate. Because dietary habits often cluster with other health-related behaviors, Model 2 is best interpreted as a conservative lifestyle-conditioned estimate rather than the total association of diet quality alone [36,37].

This study has several strengths. It is among the first to investigate the association between adherence to national dietary guidelines and glaucoma, addressing a gap in prior research, which has largely focused on single nutrients or non-country-specific diet scores. The analysis used a large, well-characterized, population-based cohort with detailed dietary assessment and objective ophthalmic measurements. The DHD-index allowed for an evaluation of overall dietary quality, capturing cumulative and synergistic nutrient effects. Additionally, the inclusion of multiple covariates—lifestyle, socioeconomic, metabolic, and ocular—allowed for rigorous confounder adjustment, enhancing the robustness of our findings.

Despite its strengths, our study has limitations. Its cross-sectional design precludes causal inferences, and residual confounding cannot be ruled out despite comprehensive adjustments. Reverse causation also cannot be excluded. However, approximately 68% of glaucoma cases in our cohort were previously undiagnosed, a proportion broadly consistent with recent European population-based data from the European Eye Epidemiology Consortium, in which 56.4% of glaucoma cases were previously undiagnosed [38]. This pattern, together with the often-prolonged asymptomatic course of glaucoma, makes major dietary changes in response to a known diagnosis less likely. Longitudinal studies are nevertheless needed to confirm these associations and to clarify the underlying biochemical and physiological mechanisms. The Maastricht Study was enriched with participants with type 2 diabetes, but glaucoma prevalence changed only modestly after exclusion of participants with type 2 diabetes, hypertension, or lipid disorders, and we found no evidence of effect modification by diabetes status. We also lacked subtype information, so our analyses pertain to overall glaucoma and cannot address potential subtype-specific heterogeneity. Finally, although glaucoma ascertainment was strengthened by specialist review of abnormal visual field together with fundus photographs and OCT scans, formal ISGEO criteria and prespecified quantitative structural thresholds (e.g., VCDR cutoffs) were not applied. Accordingly, some residual outcome misclassification cannot be fully excluded. In addition, quantitative structural OCT layer thickness measures (e.g., retinal nerve fiber layer, ganglion cell complex) were not available in our dataset and therefore could not be included in the present analysis. Future studies should explore whether diet quality is also associated with structural markers of glaucomatous neurodegeneration.

## 5. Conclusions

In conclusion, higher adherence to the Dutch Healthy Diet was associated with lower glaucoma prevalence in our study. The association changed little after accounting for IOP, but longitudinal studies are needed before any mechanistic or causal interpretation can be made.

## Figures and Tables

**Table 1:** Characteristics of glaucoma cases and non-cases.

Characteristic	No Glaucoma (N = 5171)	Glaucoma (N = 558)	*p*-Value
Age (year)	59.1 ± 8.6	63.5 ± 8.4	<0.001
Sex			
Female	2641 (51.5)	266 (47.7)	0.13
Glucose metabolism status			
Normal glucose metabolism	3441 (66.5)	293 (52.5)	
Prediabetes	769 (14.9)	100 (17.9)	<0.001
Type 2 diabetes	938 (18.1)	160 (28.7)	
Educational status			
Low	1518 (29.4)	264 (47.3)	
Middle	1481 (28.6)	124 (22.2)	<0.001
High	2172 (42.0)	209 (30.5)	
BMI (kg/m^2^)	26.5 ± 4.3	27.5 ± 4.5	<0.001
Hypertension	2559 (49.5)	344 (61.6)	<0.001
SBP (mmHg)	126.7 ± 14.9	127.3 ± 14.8	0.44
DBP (mmHg)	76.0 ± 7.6	74.7 ± 7.5	<0.001
Smoking status			
Nonsmoker	2092 (40.5)	203 (36.4)	
Former	2513 (47.9)	285 (51.1)	0.05
Current	566 (10.9)	70 (12.5)	
Alcohol consumption (g/day)	11.9 ± 13.3	11.0 ± 13.5	0.13
Physical activity (hours/weeks)	14.0 ± 8.1	13.4 ± 8.7	0.08
IOP (mmHg)	14.6 ± 4.4	16.5 ± 6.8	<0.001
Total energy intake (kcal/day)	2108 ± 598	2080 ± 606	0.30
Dutch Healthy Diet Index	84.5 ± 15.3	82.3 ± 15.8	<0.001

Values are presented as mean ± standard deviation for continuous variables and number for categorical variables. Abbreviations: N number; SBP systolic blood pressure; DBP diastolic blood pressure; IOP intraocular pressure.

**Table 2:** Characteristics of participants by adherence to the DHD (per tertiles).

Characteristic		Tertile 1 (N = 1903)	Tertile 2 (N = 1917)	Tertile 3 (N = 1909)	*p*-Value
Glaucoma		219 (11.5)	177 (9.2)	162 (8.5)	<0.001
Non-glaucoma		1684 (89.5)	1740 (90.4)	1747 (91.5)	
Age (year)		58.6 ± 9.0	59.9 ± 8.7	59.9 ± 8.4	<0.001
Female		936 (36.0)	1056 (50.1)	1433 (67.5)	<0.001
Glucose metabolism status	Normal glucose metabolism	1091 (57.3)	1236 (64.5)	1407 (73.7)	
	Prediabetes	338 (17.8)	274 (14.3)	257 (13.5)	<0.001
	Type 2 diabetes	459 (24.1)	400 (20.9)	238 (12.5)	
Educational status	Low	674 (35.4)	637 (33.2)	471 (24.7)	
	Middle	550 (28.9)	529 (27.6)	526 (27.6)	<0.001
	High	679 (35.7)	751 (39.2)	912 (47.8)	
BMI (kg/m^2^)		27.6 ± 4.5	26.7 ± 4.3	25.6 ± 3.9	<0.001
Hypertension		1087 (57.1)	989 (51.6)	827 (43.3)	<0.001
SBP (mmHg)		134.8 ± 17.3	132.8 ± 17.8	129.9 ±17.5	<0.001
DBP (mmHg)		77.0 ± 9.7	75.1 ± 9.7	73.5 ± 9.6	<0.001
Smoking status	Nonsmoker	638 (33.4)	768 (40.1)	892 (46.7)	
	Former	930 (48.9)	949 (49.5)	919 (48.1)	<0.001
	Current	338 (17.8)	200 (10.4)	98 (5.1)	
Alcohol consumption (g/day)		16.3 ± 16.4	11.4 ± 12.4	7.7 ± 8.3	<0.001
Physical activity (hours/weeks)		12.4 ± 7.9	14.2 ± 8.1	15.1 ± 8.2	<0.001
IOP (mmHg)		15.1 ± 4.9	14.8 ± 4.8	14.4 ± 4.6	<0.001
Total energy intake (kcal/day)		2264 ± 641	2075 ± 594	1977 ± 519	<0.001

Values are presented as mean ± standard deviation for continuous variables and number for cat-egorical variables. Abbreviations: N number; SBP systolic blood pressure; DBP diastolic blood pressure; IOP intraocular pressure.

**Table 3:** Association between DHD adherence, glaucoma prevalence and IOP across different models.

Panel A. Glaucoma Prevalence	Continuous	Tertile 1	Tertile 2	Tertile 3	
Model	OR (95% CI)	OR	OR (95% CI)	OR (95% CI)	P-Trend
Model 1	0.88 (0.83–0.93) **	Ref	0.66 (0.54–0.80) **	0.62 (0.50–0.76) **	<0.001
Model 2	0.95 (0.89–1.02)	Ref	0.79 (0.63–0.98) *	0.85 (0.67–1.08)	0.09
Model 3	0.89 (0.84–0.94) **	Ref	0.68 (0.56–0.83) **	0.66 (0.53–0.81) **	<0.001
**Panel** **B. IOP**	**Continuous**	**Tertile 1**	**Tertile 2**	**Tertile 3**	
**Model**	**β (95% CI)**	**β**	**β (95% CI)**	**β (95% CI)**	**P** **-** **Trend**
Model 1	−0.17 (−0.25, −0.09) **	Ref	−0.30 (−0.58, −0.03) **	−0.59 (−0.88, −0.31) **	<0.001
Model 2	−0.12 (−0.20, −0.03) *	Ref	−0.17 (−0.47, −0.13) *	−0.41 (−0.73, −0.09) *	0.006
Model 3	−0.12 (−0.20, −0.04) *	Ref	−0.21 (−0.48, −0.07) *	−0.43 (−0.72, −0.14) *	0.002

Notes: Tertiles were defined as: T1 ≤ 77.6; T2 > 77.6 to ≤ 91.2; T3 > 91.2. Tertile 1 was the reference category. For every 10-point increase in the DHD score, the corresponding odds ratios (OR) for glaucoma and beta coefficients (β) for IOP with 95% confidence intervals (CI) are presented. *p* for trend was calculated using the median DHD score for each tertile, modeled as a continuous variable. Model 1: adjusted for age, sex, and total energy intake. Model 2 (lifestyle model): Model 1 + BMI + physical activity + smoking + educational attainment. Model 3 (diseases model): Model 1 + hypertension + glucose metabolism status. Abbreviations: CI, confidence interval. * *p* < 0.05, ** *p* < 0.01.

## Data Availability

Data are available from The Maastricht Study for any researcher who meets the criteria for access to confidential data. Due to the sensitive and confidential nature of the data, they are not publicly available; the corresponding author may be contacted to request data.

## Supplementary Materials

The following supporting information can be downloaded at: https://www.mdpi.com/article/10.3390/nu18091360/s1, Figure S1: Component-specific odds prevalent glaucoma per 1-SD increase in DHD components across adjustment models; Table S1: Association of DHD adherence with glaucoma after individual adjustments for Model 2 covariates. Refs. [39,40,41,42,43] are cited in Supplementary Materials file.

## Abbreviations

The following abbreviations are used in this manuscript:

BMI	body mass index
CI	confidence interval
DASH	Dietary Approaches to Stop Hypertension
DHD-index	Dutch Healthy Diet Index
FFQ	food frequency questionnaire
IOP	intraocular pressure
MIND	DASH and Mediterranean–DASH Intervention for Neurodegenerative Delay
OR	Odds ratio
