# Supplementary material for: The Association Between Adherence to the Dutch Healthy Diet Index and Glaucoma Prevalence—The Maastricht Study
Source: Nutrients. 2026 Apr 25;18(9):1360. doi: 10.3390/nu18091360 (PMC13164943; doi:10.3390/nu18091360)
Supplement: Supplementary file 1 [file nutrients-18-01360-s001.zip › nutrients-4195064-supplementary.pdf]

# The Association Between Glaucoma Prevalence and Adherence to Healthy Diet Quality – The Maastricht Study

## Supplementary Methods

### Perimetry measurements

The Heidelberg Edge perimeter was used as a static flicker perimeter, to assess retinal sensitivity.[1] Measurements were performed in a dimly lit room by a trained examiner. Any refractive error was corrected for with external lenses. For each eye, retinal sensitivity was measured at 54 coordinates in the central and peri macular area (between 48° in the transverse plane and 42° in the sagittal plane) and results were averaged into "retinal sensitivity". In brief, the participant was instructed to fixate on a focus point and to indicate when they observed a static white light stimulus by pressing a joystick button. Light stimuli varying in strength between 0 and 35 dB and sized 0.43° in diameter (Goldmann perimeter size III) were presented on an isoluminant background of 10 cd/m$^2$. To determine the threshold of visual perception (i.e. the threshold at which the weakest presented stimulus could be perceived), we used the adaptive staircase thresholding algorithm standard automated perimetry 24-2 pattern setting. The intra-observer reliability for the assessment of the retinal sensitivity is 0.95.[1]

The device automatically classified all perimetry results (Glaucoma Hemifield Test) as: 1) within normal limits, 2) abnormally high retinal sensitivity, 3) borderline, 4) general reduction of sensitivity, and 5) outside normal limits. In addition, the device automatically calculated the following indices of measurement quality: the percentage of false positive entries, the percentage of false negative entries, and the number of fixation errors. A false positive entry indicates that the participant responded when no stimulus was presented.[2] A false negative

entry indicates that the participant did not respond to a stimulus that should be visible based on an earlier response.[2] A fixation error indicates that the fixation of the eye deviated more than 5° from the central fixation point.[2] We defined sufficient measurement quality as ⩽ 15% false positive responses.[2]

**Glaucoma Case Ascertainment**

Reader eligibility, calibration and harmonization

Five experienced glaucoma specialists were invited. Before any study grading, all completed an eligibility exercise on an external practice set after a brief calibration to standardize category definitions and decision rules. Agreement on this practice set was assessed using quadratically weighted pairwise κ. Three readers (CW, RC, MC) met the prespecified eligibility criterion and proceeded to study grading (κ > 0.60).[3] Pairwise weighted κ (estimate ± SE) were 0.81±0. 08 (CW-RC), 0.70±0.08 (RC-MC), and 0.61±0.09 (CW-MC).[3] The eligible readers then held a standards-harmonization meeting to finalize operational rules. This step follows recommendations to standardize definitions prior to grading in visual-function research.[4]

Independent grading workflow

Each visual field test was independently and masked graded by the three glaucoma specialists (CW, RC, MC) into three ordered categories (non-glaucoma, suspected glaucoma, glaucoma). The evaluation proceeded in three parts: Round 1 (independent)—agreement for 65% of cases: Round 2 (second independent pass on discordant cases)—an additional 25% reached agreement; Final consensus adjudication—the remaining 10% were resolved at a joint consensus meeting. Adjudication outcomes were used solely to create final reference labels and were not used to recompute agreement statistics.[5]

### Mediation analysis

We assessed intraocular pressure (IOP; mediator *M*) mediated the association between diet quality (Dutch Healthy Diet index; exposure *X*, modeled per 10-point increase) and prevalent glaucoma (binary outcome *Y*). We fitted (i) a linear mediator model,

$$M = \alpha_0 + \alpha_1 X + \alpha^T C + \varepsilon,$$

and (ii) a logistic outcome model,

$$\text{logit}\{P(Y = 1 | X, M, C)\} = \beta_0 + \beta_1 + \beta_2 + \beta^T C,$$

where *C* included age, sex, and total energy intake. Mediation effects (average causal mediation effect, average direct effect, total effect, and proportion mediated) were estimated using nonparametric bootstrap resampling (5 000 resamples) as implemented in the R package mediation. For interpretability, we additionally report an OR-scale coefficient-based (product-of-coefficients) approximation the log-odds scale for a 10-point higher DHD score ($\Delta X = 1$):

$$\log(OR_{indirect}) = \alpha_1 \beta_2 \Delta X,$$

$$\log(OR_{direct}) = \beta_1 \Delta X,$$

$$\log(OR_{total}) = \log(OR_{direct}) + \log(OR_{indirect}),$$

with $OR = \exp(.)$ and proportion mediated = $\log(OR_{indirect}) / \log(OR_{total})$. Percentile based 95%CI were obtained from the bootstrap distributions.

### Dietary Assessment

Dietary intake was assessed using the validated, self-administered food frequency questionnaire (Maastricht-FFQ), which was developed based on the Dutch national FFQ tool.

Dutch Healthy Diet (DHD) Index 2015 scoring: As note in the main text, the DHD15-index was developed to reflect adherence to the 2015 Dutch dietary guidelines. The detailed scoring algorithm used in the present study is provided below. The index comprises 15 components: vegetables, fruit, wholegrain products, legumes, nuts, fish, tea, dairy, fats and oils, coffee, red meat, processed meat, sugar from sweetened beverages and fruit juices, alcohol, and salt. Each component is categorized into one of five scoring types—Adequacy (A), Moderation (M), Optimum (O), Qualitative (Q), or Ratio (R)—based on its intended nutritional role. Adequacy components reward higher intake of beneficial foods (e.g., vegetables, fruit); Moderation components penalize excessive intake of harmful items (e.g., processed meat, sugary drinks); Optimum components award scores for moderate consumption (e.g., dairy); Qualitative components distinguish intake quality (e.g., filtered vs. unfiltered coffee); and Ratio components evaluate dietary fat quality (e.g., ratio of unsaturated to saturated fat). Scores were calculated using linear scaling between guideline-based minimum and maximum intake thresholds, as described previously.[6]

## Assessment of Covariates

Body weight (kg) and height (m) were assessed during a physical examination. Body mass index (BMI) was calculated as weight divided by height squared ($kg/m^2$), and used in a sensitivity analysis to evaluate whether the association between dietary adherence and glaucoma was independent of general adiposity.

Office systolic and diastolic blood pressure (mmHg) was assessed three times, from which the average was calculated.

To determine glucose metabolism status, all participants—except those using insulin—underwent a standardized 2-hour 75-gram oral glucose tolerance test after an overnight fast.

For safety reasons, participants with a fasting glucose level above 11.0 mmol/L, as determined by finger prick, did not undergo the oral glucose tolerance test.

Glucose metabolism status was classified according to the 2006 World Health Organization criteria into three categories[7]: normal glucose metabolism (NGM), prediabetes (impaired fasting glucose, impaired glucose tolerance, or both), and type 2 diabetes. Participants using glucose-lowering medication were classified as having type 2 diabetes.

## Supplementary Results

Each lifestyle-related covariate from Model 2 as added individual to Model 1 to examine its contribution to attenuation of the DHD estimate. As shown in Table S1, the association between higher DHD adherence and lower glaucoma prevalence remained statistically significant in all four analyses (OR range, 0.89-0.91). No single covariate reproduced the attenuation observed in the full lifestyle model.

**Table S1. Association of DHD adherence with glaucoma after individual adjustments for Model 2 covariates**

| Model | OR (95%CI ) |
|---|---|
| **Model 1 + BMI** | 0.89 (0.84-0.95) ** |
| **Model 1 + Physical activity** | 0.91 (0.85-0.97) * |
| **Model 1 + Somking** | 0.88 (0.83-0.94) ** |
| **Model 1 + Education** | 0.90 (0.85-0.95) ** |
| **Model 2** | 0.95 (0.89-1.02) |

Notes: * P < 0.05, ** P < 0.01
Model 1: adjusted for age, sex, and total energy intake.
Model 2 (lifestyle model): Model 1 + BMI + physical activity + smoking + educational attainment.
Abbreviations: CI, Confidence interval.

Per 1-SD higher intake, vegetable, wholegrain products, and fish were inversely associated with glaucoma odds in all three models (Model 1-3 OR ranges: vegetables 0.87 to 0.90; wholegrain product 0.80 to 0.85; fish 0.78 to 0.81). Nuts and tea also showed inverse associations in Model1 and Model 3 (nuts OR 0.85, 95%CI 0.77 to 0.94; tea OR 0.89, 95%CI 0.81 to 0.98), but not in Model 2. The DHD15 component 'Sweetened beverages and fruit juices (sugar)' was positively associated with glaucoma across Models 1-3 (OR 1.12 to 1.15). Processed meat was positive in Model 1 only and attenuated thereafter, while fruit, legumes, dairy, fats and oils, red meat showed no significant associations.

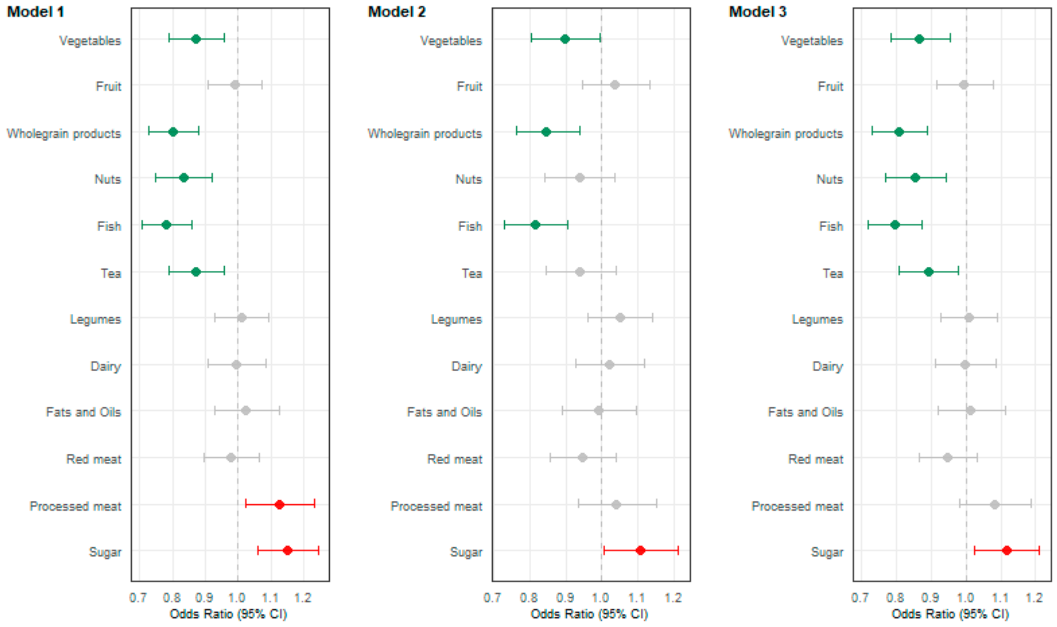


**Figure S1. Component-specific odds prevalent glaucoma per 1-SD increase in DHD components across adjustment models**

Notes: Color indicate the direction and statistical significance of associations: green, significant inverse association (OR < 1.0; two-sided P <0.05 value); red, significant positive association (OR > 1.0; two-sided P <0.05 value); gray, not statistically significant (P ⩾ 0.05).

Model 1: adjusted for age, sex, and total energy intake.

Model 2 (lifestyle model): Model 1 + BMI + physical activity + smoking + educational attainment.

Model 3 (diseases model): Model 1 + hypertension + glucose metabolism status.

Abbreviations: CI, Confidence interval. Sugar = sweetened beverages and fruit juices (DHD15 components)

## Supplementary References


1. van der Heide FCT, Mokhtar S, Khanna A, et al. Retinal Functional and Structural Neural Indices: Potential Biomarkers for the Monitoring of Cerebral Neurodegeneration: The Maastricht Study. J Alzheimers Dis. 2023;93(4):1471-1483. doi:10.3233/JAD-230104

2. Cui QN, Gogt P, Lam JM, et al. Validation and reproducibility of the Heidelberg Edge Perimeter in the detection of glaucomatous visual field defects. Int J Ophthalmol. 2019;12(4):577-581. Published 2019 Apr 18. doi:10.18240/ijo.2019.04.08

3. Cohen J. Weighted kappa: nominal scale agreement with provision for scaled disagreement or partial credit. Psychol Bull. 1968;70(4):213-220. doi:10.1037/h0026256

4. Kruger JM, Almer Z, Almog Y, et al. A Consensus Statement on the Terminology for Automated Visual Field Abnormalities. J Neuroophthalmol. 2022;42(4):483-488. doi:10.1097/WNO.0000000000001622

5. Kottner J, Audigé L, Brorson S, et al. Guidelines for Reporting Reliability and Agreement Studies (GRRAS) were proposed. J Clin Epidemiol. 2011;64(1):96-106. doi:10.1016/j.jclinepi.2010.03.002

6. Looman M, Feskens EJ, de Rijk M, et al. Development and evaluation of the Dutch Healthy Diet index 2015. Public Health Nutr. 2017;20(13):2289-2299. doi:10.1017/S136898001700091X

7. World Health Organization (2006): Definition and diagnosis of diabetes mellitus and intermediate hyperglycemia: report of a WHO/IDF consultation. Geneva, Switzerland.
